# Dynamic Lumbosacral Magnetic Resonance Imaging in a Dog with Tethered Cord Syndrome with a Tight Filum Terminale

**DOI:** 10.3389/fvets.2017.00134

**Published:** 2017-08-18

**Authors:** Steven De Decker, Vicky Watts, David M. Neilson

**Affiliations:** ^1^Department of Clinical Science and Services, Royal Veterinary College, University of London, Hatfield, United Kingdom

**Keywords:** spinal dysraphism, spinal malformation, magnetic resonance imaging, cauda equina, conus medullaris

## Abstract

A 1-year and 11-month- old English Cocker Spaniel was evaluated for clinical signs of progressive right pelvic limb lameness and urinary incontinence. Neurological examination was suggestive of a lesion localized to the L4–S3 spinal cord segments. No abnormalities were seen on magnetic resonance imaging (MRI) performed in the dog in dorsal recumbency and the hips in a neutral position and the conus medullaris ended halfway the vertebral body of L7. An MRI of the hips in extended and flexed positions demonstrated minimal displacement of the conus medullaris in the cranial and caudal directions, respectively. Similar to the images in neutral position, the conus medullaris ended halfway the vertebral body of L7 in both the extended and flexed positions. In comparison, an MRI of the hips in neutral, extended, and flexed positions performed in another English Cocker Spaniel revealed obvious cranial displacement of the conus medullaris with the hips in extension and caudal displacement with hips in flexion. A standard dorsal lumbosacral laminectomy was performed. Visual inspection of the vertebral canal revealed excessive caudal traction on the conus medullaris. After sectioning the distal aspect of the filum terminale, the conus medullaris regained a more cranial position. A neurological examination 4 weeks after surgery revealed clinical improvement. Neurological examinations at 2, 4, 7, and 12 months after surgery did not reveal any abnormalities, and the dog was considered to be clinically normal. Tethered cord syndrome with a tight filum terminale is a very rare congenital anomaly and is characterized by an abnormally short and inelastic filum terminale. Therefore, this disorder is associated with abnormal caudal traction on the spinal cord and decreased physiological craniocaudal movements of the neural structures within the vertebral canal. Although further studies are necessary to evaluate and quantify physiological craniocaudal movement of the spinal cord and conus medullaris in neurologically normal dogs, the results of this report suggest further exploration of dynamic MRI to demonstrate decreased craniocaudal displacement of the conus medullaris in dogs with tethered cord syndrome with a tight filum terminale.

## Case Presentation

**Figure 1:**
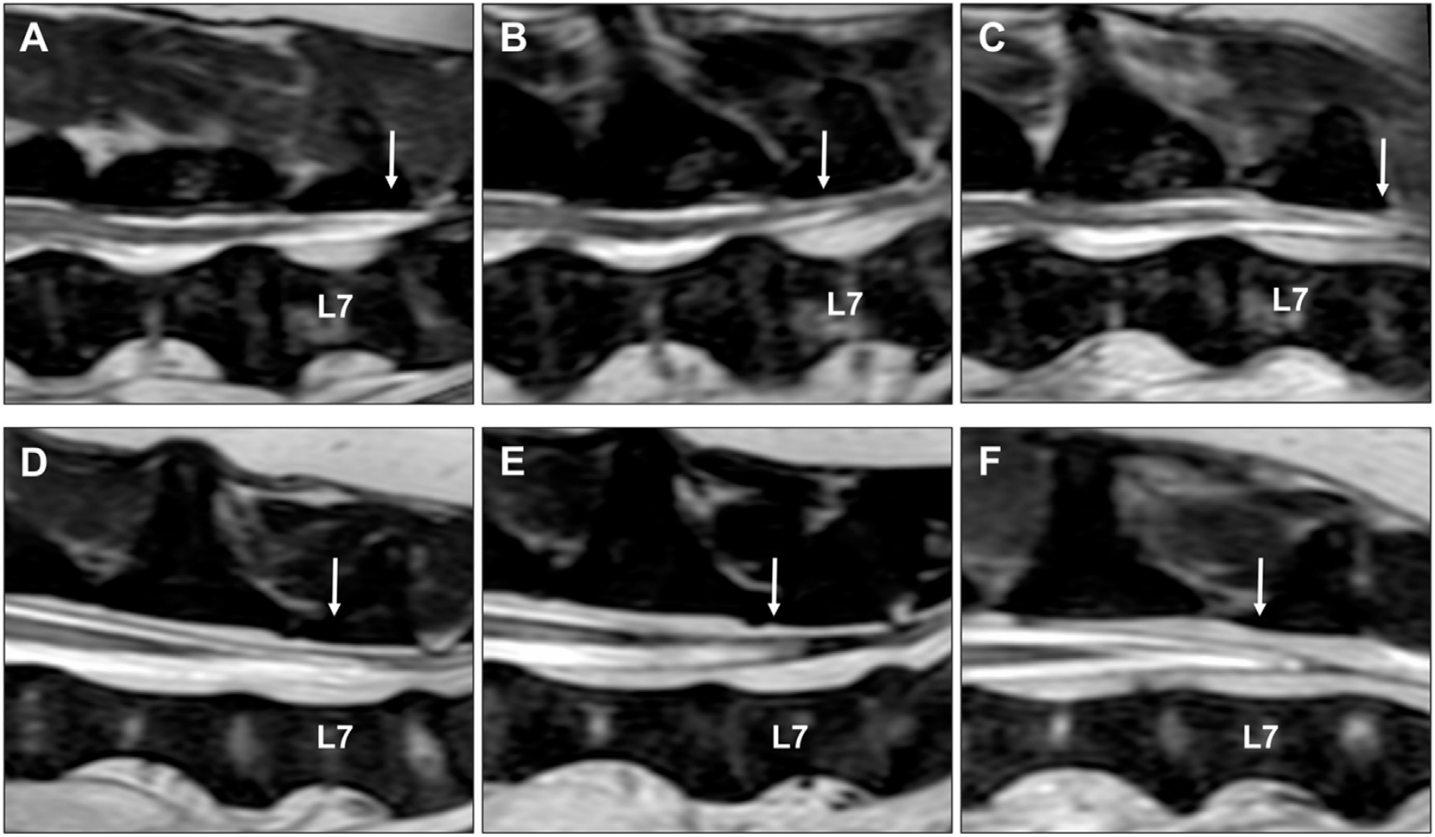
Sagittal T2-weighted images of an English Cocker spaniel with thoracolumbar intervertebral disk extrusion with the hips in neutral **(A)**, extended **(B)**, and flexed **(C)** positions. **(A)** The conus medullaris (arrow) terminates at the caudal aspect of the L7 vertebral body. **(B)** The conus medullaris (arrow) is displaced in a cranial direction when the hips are extended and terminates now halfway the L7 vertebral body. **(C)** The conus medullaris (arrow) is displaced in a caudal direction when the hips are flexed and terminates now at the level of the L7–S1 intervertebral disk space. Sagittal T2-weighted images of an English Cocker spaniel with tethered cord syndrome with a tight filum terminale with the hips in neutral **(D)**, extended **(E)**, and flexed **(F)** positions. The conus medullaris (arrow) terminates halfway the L7 vertebral body in all three positions, and only minimal displacement is seen between different positions.

A 1-year and 11-months-old, female neutered English Cocker Spaniel was evaluated for clinical signs of progressive right pelvic limb lameness of 16 months duration and intermittent urinary incontinence of 2 weeks duration. Orthopedic and neurological examinations; radiographs of the hips, pelvis, and right stifle; and spinal magnetic resonance imaging (MRI) performed at 10 months before referral had not revealed the underlying cause of the dog’s clinical signs. No clinical improvement was seen after medical treatment with carprofen and meloxicam. General physical examination did not reveal any abnormalities. Neurological examination revealed mild paraparesis, right pelvic limb lameness, a low-tail carriage, decreased tail tone, a decreased withdrawal reflex in the right pelvic limb, and proprioceptive deficits in the right pelvic limb expressed by delayed hopping, but intact paw placement. Pain could be elicited on lumbosacral palpation, dorsal extension of the tail, and extension of both hips. No other neurological deficits were identified. Her neurological lesion was localized to the L4–S3 spinal cord segments. A complete blood count, biochemistry panel, and urinalysis, including bacterial culture, did not reveal any abnormalities. After premedication with methadone (0.2 mg/kg IM) and acepromazine (0.01 mg/kg IM), anesthesia was induced with propofol (4–6 mg/kg, IV) and maintained with sevoflurane in 100% oxygen. An MRI (1.5 T, Intera, Philips Medical Systems, Eindhoven, the Netherlands) of the lumbar and lumbosacral vertebral column was performed with the dog supported in dorsal recumbency by a high-density foam trough with flexed limbs in a neutral position (i.e., frog-leg position). The imaging protocol included sagittal, transverse, and dorsal plane T2-weighted [repetition time (ms) (TR), echo time (ms) (TE), 3,000/120], sagittal and dorsal plane T2-weighted short tau inversion recovery (STIR) (TR/TE, 3,612/80), and transverse plane T2-weighted BAL TGRAD (TR/TE, 7.9/3.9) sequences. Sagittal and transverse plane T1-weighted (T1W TSE) (TR/TE, 400/8) images were acquired before and after IV injection with gadolinium contrast (0.1 ml/kg gadoterate meglumine, Dotarem, Guerbet, Milton Keynes, England). No abnormalities were seen on MRI, and the conus medullaris ended halfway the vertebral body of L7 (Figure 1). Sagittal T2-weighted images were subsequently acquired with the hips in extended and flexed positions. Extended views were obtained by extending the pelvic limbs caudally and securing them in maximal extension of the coxofemoral joints with velcro straps secured to the MRI table. Flexed views were obtained by pulling the pelvic limbs cranially and restraining them beside the thorax with extended stifles. An MRI with the hips in extended and flexed positions demonstrated minimal displacement of the conus medullaris in the cranial and caudal directions, respectively. Similar to the images in neutral position, the conus medullaris ended halfway the vertebral body of L7 in both the extended and flexed positions (Figure 1). However, the dog demonstrated an increase in heart rate, from 60 to 90 beats per minute, when images were acquired with the lumbosacral joint in flexed position. The heart rate returned to normal level after administration of an IV bolus of 0.05 mg/kg methadone. Differential diagnoses included tethered cord syndrome with tight filum terminale and dynamic lumbosacral vertebral canal stenosis. Recovery from general anesthesia was uneventful, and a neurological examination before discharge from hospitalization did not reveal any deterioration of clinical signs or neurological deficits.

For comparative purposes, an MRI of the hips in neutral, extended, and flexed positions was performed on the same day in a 6-year and 4-month-old, male neutered, English Cocker Spaniel with non-ambulatory paraparesis caused by a lesion affecting the T3–L3 spinal cord segments. An MRI revealed generalized intervertebral disk degeneration and an L1–L2 intervertebral disk extrusion as the cause of the dog’s clinical signs. With the hips in neutral position, the conus medullaris ended at the caudal aspect of the L7 vertebral body (Figure 1). The conus medullaris moved cranially until halfway the vertebral body of L7 in the extended position and moved caudally overlying the L7–S1 intervertebral disk space when an MRI was performed with the hips in a flexed position (Figure 1).

**Figure 2:**
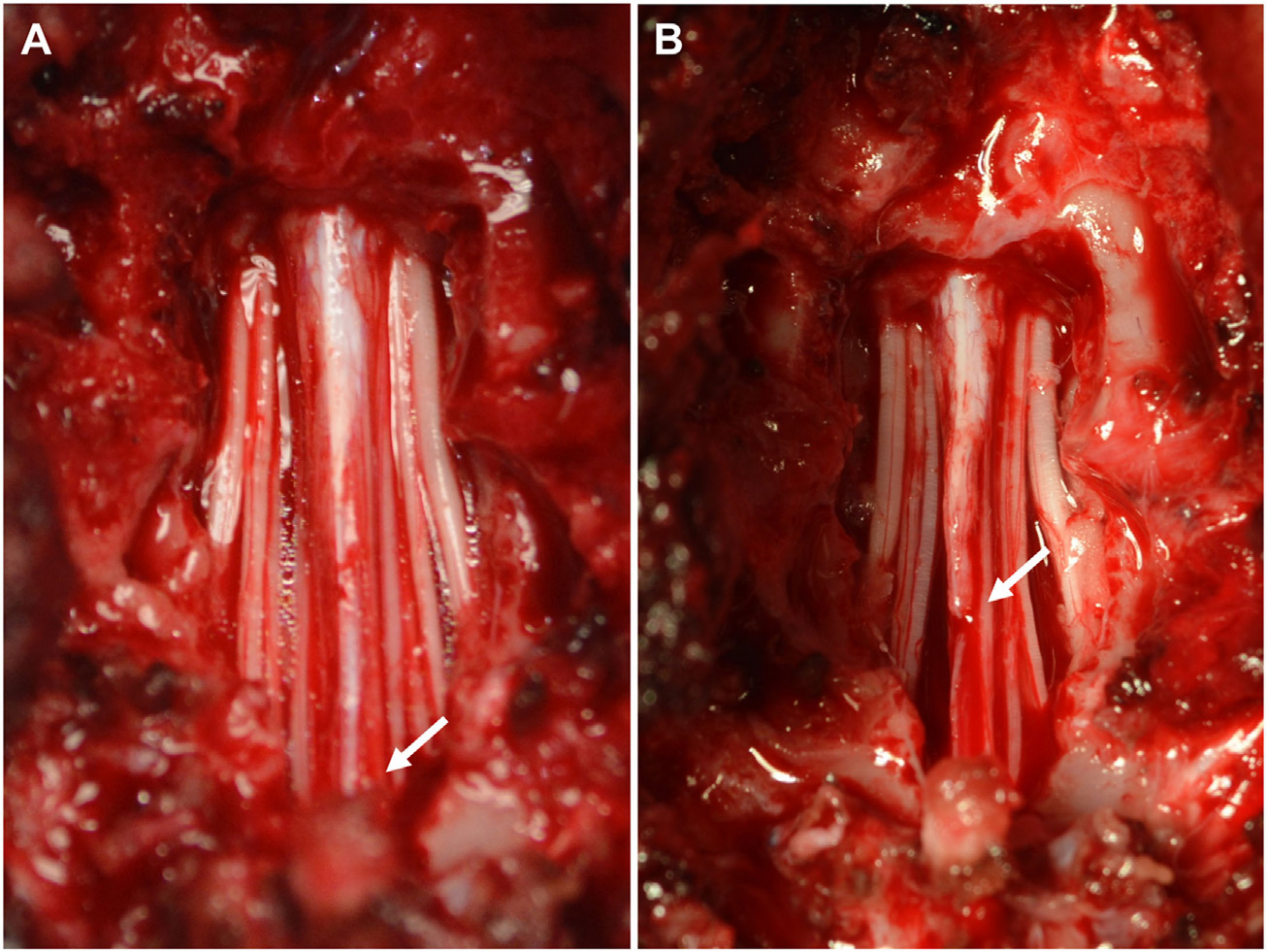
Intraoperative pictures before **(A)** and after **(B)** sectioning the filum terminale. **(A)** Visual inspection of the vertebral canal reveals excessive caudal traction on the conus medullaris (arrow). **(B)** The conus medullaris regains a more cranial position after sectioning the distal aspect of the filum terminale.

Medical management was started with gabapentin (10 mg/kg, q8h, PO) and restricted exercise. A re-examination 2 weeks later demonstrated progression of her clinical signs characterized by more severe urinary incontinence. General anesthesia was induced and maintained with the aforementioned protocol. A standard dorsal lumbosacral laminectomy, from L7 to S1 was performed. Subjective visual inspection of the vertebral canal revealed excessive caudal traction of the conus medullaris. The conus medullaris was also not as freely moveable in the vertebral canal as normally expected. No other abnormalities were detected. After sectioning the distal aspect of the filum terminale from the cranial aspect of the lamina of S2, the conus medullaris regained a more cranial position (Figure 2). The wound was closed routinely. Intraoperative analgesia was provided with ketamine (loading dose of 0.5 mg/kg IV followed by infusion at a rate of 10 μg/kg/min IV) and methadone (0.1 mg/kg q4h, IV). Postoperative analgesia consisted of a combination of methadone (0.2 mg/kg, q4h, IV), carprofen (2 mg/kg, q12h, PO), and gabapentin (10 mg/kg, q8h, PO). The dog was discharged from the hospital 2 days after surgery. The owner was advised to ensure strict rest for 4 weeks in combination with gabapentin and carprofen for 2 more weeks. The surgical findings were considered diagnostic for tethered cord syndrome with a tight filum terminale. The results of a neurological examination 4 weeks after surgery revealed improvement of the dog’s signs. At this time, the dog demonstrated mild lameness and proprioceptive deficits in the right pelvic limb. The urinary incontinence had resolved. Neurological examinations at 2, 4, 7, and 12 months after surgery did not reveal any abnormalities, and the dog was considered clinically normal.

## Background

Tethered cord syndrome is a rare congenital anomaly in which progressive neurological signs are caused by abnormal caudal traction on the conus medullaris and caudal spinal cord segments (1, 2). This anomaly can be seen in association with a variety of spinal malformations and has been considered a form of occult spinal dysraphism (3). However, in the majority of human patients, tethered cord syndrome is associated with an abnormal short, thickened, and inelastic filum terminale without other spinal malformations (3). This is also referred to as tethered cord syndrome with a tight filum terminale (1, 4) and has been reported only once previously in the veterinary literature (5). As a consequence of excessive caudal traction, tethered cord syndrome is classically associated with an abnormal caudodorsal position of the conus medullaris (1–4). In people, the tip of the conus medullaris terminates at the level of L1, and termination caudal at the L2 vertebral level in patients with suggestive clinical signs is considered diagnostic for tethered cord syndrome with a tight filum terminale (1–3). However, making a diagnosis of tethered cord syndrome in dogs is challenging because of variation in termination of the conus medullaris (6) and filum terminale (7) between, and possibly also within, dog breeds. Obtaining a diagnosis is further complicated by the fact that a proportion of people with tethered cord syndrome presents with the conus medullaris in an anatomical normal position without evidence of caudal displacement. This is referred to as occult tethered cord syndrome, and development of clinical signs has been attributed to decreased elasticity of the filum terminale and hence increased traction on the conus medullaris (8, 9). Decreased elasticity of the filum terminale has been suggested to limit the normal physiologic movements of the spinal cord and conus medullaris in response to everyday movements of the body and vertebral canal (10). Performing MRI in different body positions (i.e., the prone and supine position) has therefore been considered with the aim to demonstrate decreased conus medullaris motion in people with occult tethered cord syndrome (9).

## Discussion

Obtaining a diagnosis of tethered cord syndrome with a tight filum terminale is challenging in dogs for several reasons (5). The anatomical variation in conus medullaris termination and the theoretical possibility of occult tethered cord syndrome make it difficult to consider an abnormal caudal position of the conus medullaris as a reliable diagnostic criterion in dogs (5). Therefore, this case report explored the possibility of dynamic MRI with the hips in neutral, extended, and flexed positions to facilitate diagnosing this rare congenital anomaly. Although the results of single-case reports should be interpreted with caution, our findings suggest that dynamic MRI might be useful in evaluating the degree of craniocaudal displacement of the conus medullaris and thereby indirectly assessing the elasticity of the filum terminale. Compared to a dog of the same breed and imaged under identical conditions, the dog with tethered cord syndrome demonstrated only minimal craniocaudal displacement of the conus medullaris after an MRI was performed with the hips in extension and flexion (Figure 1). This finding in combination with suggestive clinical signs and exclusion of other structural abnormalities were considered suggestive for tethered cord syndrome with a tight filum terminale. Excessive caudal traction on the conus medullaris was confirmed during surgery, and the dog recovered completely after sectioning of the filum terminale. Although sectioning of the filum terminale is considered a technically easy and relative safe surgical procedure, reported complications in humans include cerebrospinal fluid leakage, headaches, infection, pseudomeningocele, and retethering (11). Ideally, a second MRI scan would have been performed to evaluate a potential increase in craniocaudal conus medullaris displacement after sectioning of the filum terminale. However, this was not performed because there was no clinical indication to justify the costs and requirement of general anesthesia in this clinical case.

Craniocaudal displacement of the conus medullaris in response to physiological movements of the vertebral canal and extremities has been well documented in humans (12–14). It has been suggested that the sliding or craniocaudal movement of the neural structures within the vertebral canal should be considered a protective mechanism, which represents transmission of tensile forces through the neural structures, thereby preserving the spinal cord and nerve roots from excessive strain (14). Therefore, maintaining free sliding of the neural structures and meninges has been considered an essential condition for maintaining a healthy and normal functioning spinal cord (13, 14).

The conus medullaris is the most caudal tapered ending of the spinal cord. This is continued by a filamentous structure, the filum terminale, which extends caudally and attaches to a sacral or caudal vertebra (7). The normal filum terminale has elastic properties and has therefore the capability of protecting the spinal cord by buffering linear stress to the spinal cord during physiological flexion and extension of the vertebral canal (15). Tight filum terminale is characterized by shortening and loss of elastic properties, which is associated with decreased transmission of tensile forces and restricted spinal cord movement (15, 16). This decreased elasticity will result in repetitive, excessive, and progressive traction on the conus medullaris and caudal spinal cord segments. The cumulative effects of hypoxia, decreased oxidative metabolism, and anatomic deformation will then ultimately result in progressive signs of pain and dysfunction of the caudal lumbar and lumbosacral spinal cord segments (17, 18).

In human medicine, several physical tests have been described to facilitate the clinical diagnosis of radiculopathies and spinal cord disorders (19). One of these physical tests is the straight leg raise test in which the patient is placed in dorsal recumbency and asked to flex the hips, while keeping the knees in an extended position (12, 19). This position is similar to the position of the dog, described in this report, when lumbosacral MR images were obtained with flexed hips and extended stifle joints. In agreement with the findings in the “control” dog, a caudal displacement of the conus medullaris is observed in asymptomatic people after performing the straight leg raise test (12–14). The importance of extending the stifle joint while flexing the hips is demonstrated by a lack of caudal conus medullaris displacement when both the hips and knees are flexed. This latter position is referred to as the sham straight leg raise test (14).

**Figure 3:**
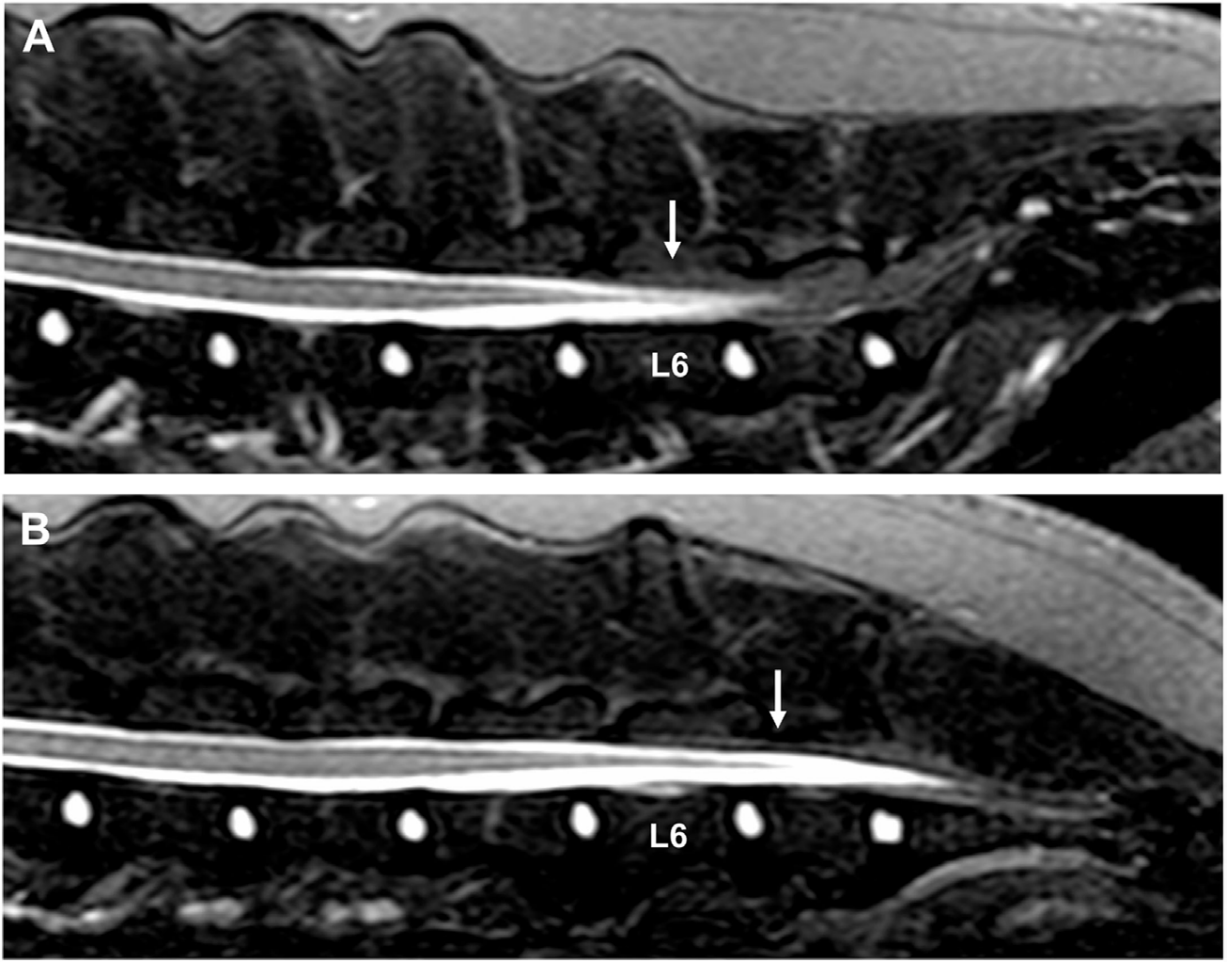
Sagittal short tau inversion recovery images of a 3-year-old Bullmastiff with caudal lumbar spinal hyperesthesia. **(A)** The conus medullaris terminates at the caudal aspect of the L6 vertebral body with the hips in a neutral position (arrow). **(B)** The conus medullaris is displaced caudally and terminates now at the cranial aspect of the L7 vertebral body when the hips are flexed (arrow). The MRI study did not reveal the cause of the dog’s clinical signs.

Although the findings of this report suggest that dynamic MRI can be considered to evaluate the presence or absence of physiologic craniocaudal conus medullaris displacement, it is difficult to draw firm conclusions from single-case reports. The variation of conus medullaris termination between and possibly within breeds makes it difficult to objectively quantify the normal degree of conus medullaris displacement in unaffected dogs. In humans, the degree of hip flexion has also been associated with differences in magnitude of conus medullaris displacement, and therefore, variations can be seen between and within individual patients (13). Although a diagnosis of tethered cord syndrome with a tight filum terminale was suspected based on the subjective observation of decreased to absent conus medullaris displacement between extended and flexed positions, further studies are necessary to evaluate if this finding can be used as a reliable diagnostic criterion for this rare congenital disorder. It is further unclear which MRI sequence would be most useful to evaluate termination of the conus medullaris. The presence of a large quantity of epidural fat in the lumbosacral vertebral canal can complicate recognizing the most caudal tip of the conus medullaris on conventional T2-weighted and T1-weighted images. Applying fat suppression techniques, such as fat saturation techniques, STIR, spectral attenuated inversion recovery (SPIR), or spectral attenuated inversion recovery sequences (SPAIR) can therefore be considered when trying to evaluate termination of the conus medullaris (Figure 3).

## Concluding Remarks

This case report explored the possibility of dynamic MRI with the hips in neutral, extended, and flexed positions to evaluate the degree of craniocaudal displacement and indirectly the elastic properties of the filum terminale. Decreased to absent displacement of the conus medullaris was considered suggestive for decreased elasticity of the filum terminale, and hence, a diagnosis of tethered cord syndrome with a tight filum terminale was suspected. Although this diagnosis was confirmed during surgery, further studies are necessary to evaluate craniocaudal conus medullaris displacement in neurologically affected and unaffected dogs before this finding can be considered a reliable diagnostic criterion for tethered cord syndrome with a tight filum terminale in dogs.

## Ethics Statement

The owners of clinical cases described in this study gave informed consent for the diagnostic procedures, treatment, and use of clinical data, such as medical history, imaging studies, and intraoperative pictures for research and publication purposes. All owners were informed that dynamic MRI with the hips in neutral, extended, and flexed positions would be performed. Because all diagnostic studies and initiated treatments were part of daily clinical activities, this project did not reach the threshold for submission to the local ethical and welfare committee.

## Author Contributions

SD, VW, and DN were involved in the care of this patient and were involved in planning this case report. SD was responsible for conception of this case report and preparation of the first draft. VW and DN provided feedback and were involved in revising the manuscript. All authors have read and approved the final version of the manuscript.

## Conflict of Interest Statement

The authors declare that the research was conducted in the absence of any commercial or financial relationships that could be construed as a potential conflict of interest.
